# Supplementary material for: Western diet-induced shifts in the maternal microbiome are associated with altered microRNA expression in baboon placenta and fetal liver
Source: Front Clin Diabetes Healthc. 2022 Sep 8;3:945768. doi: 10.3389/fcdhc.2022.945768 (PMC10012127; doi:10.3389/fcdhc.2022.945768)
Supplement: Supplementary file 1 [file DataSheet_1.pdf]

Supplementary Material

Western diet-induced maternal microbial dysbiosis is associated with steatosis and altered expression of microRNAs in baboon fetal liver and placenta

Kameron Y. Sugino[1,†], Ashok Mandala[1,†], Rachel C. Janssen[1], Sunam Gurung[2], MaJoi Trammell[3], Michael W. Day[3], Richard S. Brush[4], James F. Papin[5], David W. Dyer[3], Martin-Paul Agbaga[4,6], Jacob E. Friedman[1,7], Marisol Castillo-Castrejon[7,8], Karen R. Jonscher[1,9,*] and Dean A. Myers[2]

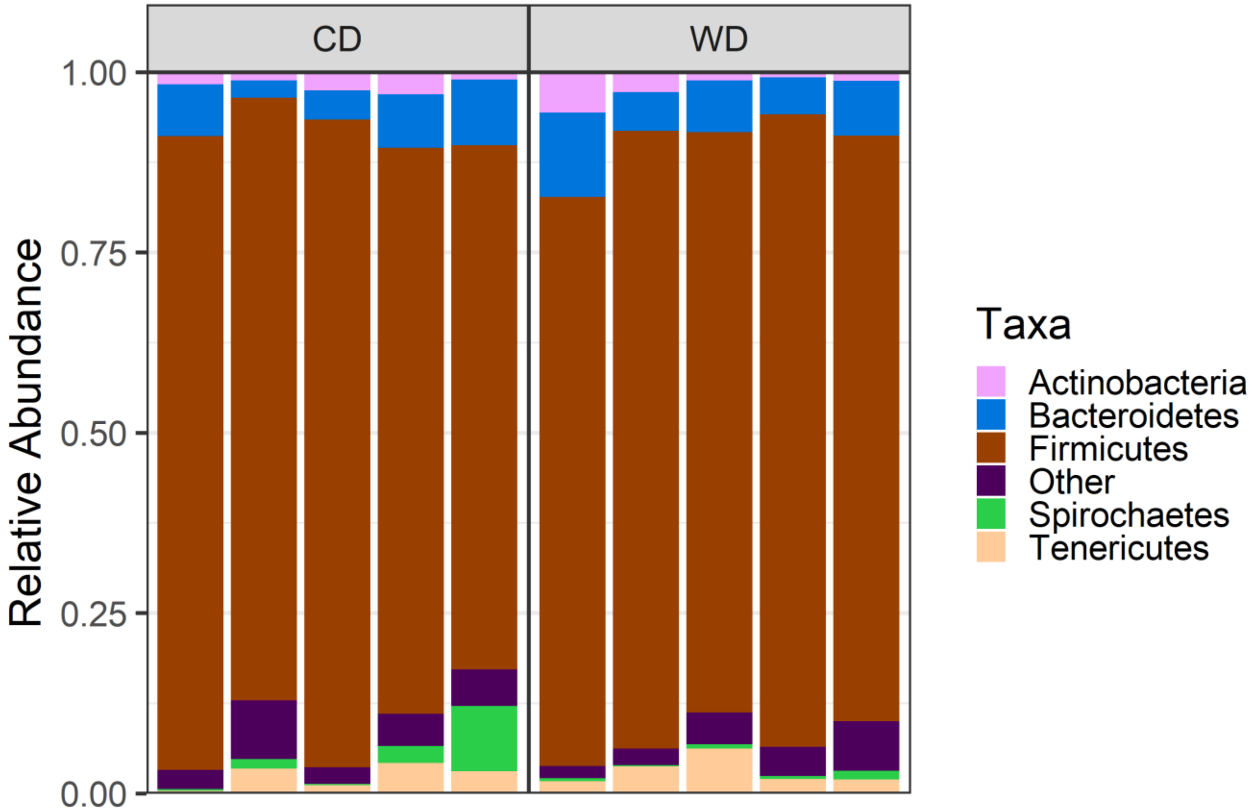

Supplementary Figure S1. Microbial abundances of the top five most abundant phyla for each feces sample. The 15 lower abundant phyla are displayed as “Other”.

Supplemental Table S1. qPCR primer sequences for mRNA expression.

Gene	Forward Primer	Reverse Primer
ACACA	AGCCCTCAACAAAGTCCTCG	AGTGGGTCACCCCATTGTTG
ACTA2	GGCAAGTGATCACCATCGGA	GTGGTTTCATGGATGCCTGC
ACTB	GGGAAATCGTGCGTGACATT	AGGTAGTTTCGTGGATGCCA
CCR2	TTGACGTGAAGCAAATCGGG	CCAGCATGTTGCCCACAAAA
COL1A1	AAGGACAAGAGGCACGTCTG	CAGGAAGGTCAGCTGGATGG
COL3A1	GGTCCAAAGGGTGACAAGGG	GGCCAGGAGGACCAATAGGA
FAP	AGTTTCAGCGACTACGCCAA	TCTTGATCAGTGCGTCCGTC
FASN	GAGCACAACAGGGTGCTAGA	TTGATGATCAGGTCCACGGC
HMOX1	AGCAACAAAGCGCAAGACTC	GATCCATCGGAGAAGCGGAG
ICAM1	CAGTTCGTGCTGAAAGCCAC	ATCGGGGTCCATACAGGACA
IL10	CTGAGAACCACGACCCAGAC	GAAGAAATCGATGACAGCGCC
IL12B	GCAGCTGGTCATCTCTTGGT	CACCATTTCTCCAGGGGCAT
IL1B	GCTCTCCACCTCCAGGGACAGG	TGAGGCCCAAGGCCACAGGT
IL1B *	TGAAAGCTCTCCACCTCCAG	TTGGGCAGACTCGAATTCCA
IL6	CAGTTCCTGCAGAAAAAGGCAA	GAGATGCGTCGTCATGTCCT
IL6 *	ATGCAATAACCACCCCTGAA	CTGCAGCCACTGGTTCTGT
IL8 *	CCTTTCCACCCCAAATTTATC	TTCTGTATTGACGCAGTGTGG
MCP1 (CCL2)	TTCTGTGCCTGCTGCTCATAG	GGGGGCATTGATTGCATCTGG
NCF4	ATACCTGCCCTCAACGCCTA	CGGGGACACACTCTTGACTT
NFE2L2 (NRF2)	TCTGCCAACTACTCCCAGGT	AACGTAGCCGAAGAAACCTCA
NOS2	AAGACCAGGCTGTCGTTGAG	GGATTCTGCAGCTGAGTGGT
PDGFA	AAGCAGCCAACCAGATGTGA	AGACCGCACACTGGCAATAA
RPS9	TTGAAGCTGATCGGCGAGTAT	TCTCATCAAGCGTCAGCAGTT
SREBF1 (SREBP1)	GGATTGCACGTTCGAAGACAT	AGAGAGGAGCTCAGTGTGGT
TGFB1	GAGCCCTGGACACCAACTAC	GAGGTCCTTGCGGAAGTCAA
TLR4	GCTTCCTCCGTTTTCCAGAACTGC	TGGAGAGGTGGCTTAGGCTCTGA
TNF	TCGAACCCCAAGTGACAAGCCT	GCCATTGGCCAGGAGGGCAT
TNF *	TTCAGCTGGAGAAGGGTGAT	CCAAAGTAGACCTGCCCAGA
TNFSF12	CAGGACCCATCGGAACTGAA	CCGTGTTTTCCGGCCTTTAG
TREM2	ATCTACAACCCCACGATGCG	CAGAGATCTCCAGCATCCCG
VCAM1	TTTTTGTCAATGTTGCCCCC	CAGGCTGTAGCTCCCCATTAG
VEGFA	CCCACTGAGGAGTCCAACAT	CTCCTATGTGCTGGCCTTGG

* Denotes qPCR primer set used in placental tissue.

Supplemental Table S2. Differential expression levels of inflammatory genes in fetal liver.

Gene	Fold change	*P* value	Comment
TNF			Undetectable amplification
CCL2	1.090	0.707	
TLR4	0.888	0.630	
NOS2			Undetectable amplification
IL10	1.391	0.486	
TGFB1	1.228	0.451	
PDGFA	1.240	0.364	
IL12B	1.156	0.733	

Supplementary Table S3. Tests of the *Firmicutes*/*Bacteroidetes* ratio with diet group and maternal parameters.

Variable	Value	*P* value
Diet group*	15	0.6905
Body weight	0.43030	0.2145
SSF	0.24316	0.4984
CRP	-0.33333	0.3807
Triglycerides	-0.11667	0.7650
Fasting glucose	0.53333	0.1392
Fasting insulin	-0.35758	0.3104
HDL	-0.40606	0.2443
LDL/VLDL	-0.24268	0.5292
Days gestation	0.16035	0.6803
Maternal weight at delivery	0.53333	0.1392
Placental weight	0.22893	0.5855

Variables analyzed by Spearman’s correlation and rho value shown (excluding diet group).
*Association analyzed by Wilcoxon rank sum test.
CRP, C-reactive protein; SSF, sum of skin folds.
